# Antimicrobial Activity and Wound-Healing Capacity of Birch, Beech and Larch Bark Extracts

**DOI:** 10.3390/molecules27092817

**Published:** 2022-04-28

**Authors:** Stefanie Emrich, Anja Schuster, Thomas Schnabel, Gertie Janneke Oostingh

**Affiliations:** 1 Biomedical Sciences, Salzburg University of Applied Sciences, Urstein Sued 1, 5412 Puch, Austria; s.emrich@gmx.at (S.E.); geja.oostingh@fh-salzburg.ac.at (G.J.O.); 2 Department of Forest Products Technology & Timber Constructions, Salzburg University of Applied Sciences, Markt 136a, 5431 Kuchl, Austria; thomas.schnabel@fh-salzburg.ac.at; 3 Faculty of Furniture Design and Wood Engineering, Transilvania University of Brasov, B-dul. Eroilor nr. 29, 500036 Brasov, Romania

**Keywords:** antimicrobial activity, bark extract, birch, beech, larch, wound healing

## Abstract

Bark is a major by-product of woodworking industries. The contents of several wood species are known to harbor antimicrobial, antiviral, anti-inflammatory and wound-healing capacities. The aim of this work was to identify beneficial properties of Austrian larch, birch and beech bark extracts for their potential usage as additives or active ingredients in dermatological applications. Bacterial agar diffusion assay and resazurin-based broth microdilution assay were used to evaluate anti-bacterial activity. To gain more insight into the cellular response to bark extracts, viability-, scratch-assays and ELISAs were performed. Birch and beech extracts showed strong antimicrobial activities against Gram-positive bacteria, including *Cutibacterium acnes, Staphylococcus epidermidis* and MRSA. Wound closure was enhanced with birch and beech extracts as compared to controls in the scratch-assays. Whereas beneficial properties of birch bark components have previously been described, the similar effects of beech extracts are novel. The combined positive effect on wound-healing and antimicrobial activity has great potential for the treatment of various skin diseases, including acne in future dermal applications.

## 1. Introduction

According to the Association of Austrian Timber Industry, wood trade is a key sector of the Austrian economy. Woodworking companies generate products in the building-, furniture-, sawmill- and ski-industries worth around EUR 8.03 billion a year [1]. Beech (*Fagus sylvatica L.*) is the most prevalent deciduous tree species in Europe with the highest value for producing furniture [2,3]. Birch (*Betula pendula Roth* and *Betula pubescens*) harbors, country dependent, up to 15% of the hardwood standing volume in Western Europe. Birch trees tolerate variable climates and colonize forest gaps quickly, contributing to forest resilience and biodiversity. Therefore, birch wood is an attractive and valuable source for building and furniture sectors [4]. Larch (*Larix decidua*) is native to the Alps and Caucasian mountains and it forms extensive forests at high altitudes. In the building industry, it is valued due to its tough, waterproof and durable qualities.

It is already known that various wood species contain substances of anti-bacterial activity and many of these compounds are present in the bark of the trees [5,6,7,8,9,10], quite likely to protect it from pathogenic microorganisms in its natural habitat. Since wood bark is a completely biological major byproduct of wood industries, its usage would be a great opportunity for an eco-friendly, resource-saving economy.

Secondary plant metabolites, such as aliphatic compounds including terpenes, terpenoids, fatty acids and phenolic compounds (i.e., flavonoids, simple phenols or tannins) are valuable biochemicals. Phenolic compounds play a major role in the constitutive as well as the inducible defense of various trees against pathogens [11]. In this study, the focus was placed on the potential use of certain bark extracts for dermatological application in terms of antimicrobial activity in common skin-related bacteria, wound-healing and immune-regulating capacities. The incidence of *Staphylococcus aureus* in Western European countries has more than doubled in the past 25 years. The most common site of *S. aureus* infection is the skin, but any tissue can be infected by the pathogen [12]. In addition to *S. aureus*, methicillin-resistant *S. aureus* represents a major part of bacterial infections in industrialized countries due to its diminished sensitivity to antibiotics treatment [13]. *Cutibacterium acnes* is known to play a major part in the inflammatory skin condition of acne, which affects up to 90% of all teenagers. The virulent bacteria in acne lesions show an increased resistance to several antibiotics such as clindamycin, erythromycin and tetracyclines [14,15].

In addition to the antimicrobial capacities, certain bark extracts harbor wound-healing capacities as well as antiviral and anti-inflammatory properties [16,17,18,19]. The wound healing capacities are mainly related to the compound betulin, a triterpene found in birch bark; however, information on other tree species, especially in terms of wound healing, is in large parts missing.

The combined properties of antimicrobial activity and wound-healing capacities make bark extracts extremely attractive for various applications on the human skin. There is a great potential for these extracts in numerous treatments in dermatology, including acne vulgaris, and may they prevent small superficial skin injuries from infection and support the healing process. However, a prerequisite for the use of these extracts in dermatological applications is the use of biocompatible solvents during the various extraction techniques. So far, a number of studies have used methanol as a solvent for extracting polyphenols, and DMSO and glycerin have been used for extracting wood and bark compounds [6,8,20]. 

The aim of this work was to identify beneficial properties of larch, birch and beech bark extracts in terms of antimicrobial activity and wound-healing capacity for the use of these bark extracts as additives or active ingredients in dermatological applications in cosmetics and medicine. The data showed that biocompatible birch as well as beech bark extracts contain a number of biomolecules with antimicrobial activity against a range of skin-related pathogens and properties that facilitate wound healing in vitro.

## 2. Materials and Methods

### 2.1. Bark Extracts

Bark extracts were obtained from birch (*Betula pendula*), beech (*Fagus sylvatica*) and larch trees (*Larix decidua*). To extract compounds, the bark (125 g bark/L) was heated in water to 200 °C using a pressure vessel for 3 h (0.8 bar), then dried to powder and diluted in H_2_0 to a stock solution of 10 mg/mL. Extracts were centrifuged 3000× *g* for 10 min at room temperature (RT), filtered with a 0.45 µm filter unit (Merck MILLIPORE, Burlington, MA, USA) and stored at 4 °C for short term storage (up to 1 month) and −20 °C for long term storage. 

### 2.2. Total Phenolic Content

To determine the total phenolic content of different bark extracts, the Folin–Ciocalteu method was used. The diluted concentration of 1 mg/mL (solid content) in a volume of 200 µL was filled in a test tube and mixed with 500 µL of the Folin–Ciocalteu reagent and 3 mL deionized water. The mixture was shaken and incubated for 3 min before 2 mL of 20% (*w*/*v*) sodium carbonate was added. The mixture was incubated for 1 h protected from light, then absorbance was measured with a spectrophotometer (Shimadzu UV mini 1240 UV/VIS) at a wavelength of 756 nm. Results were calculated from triplicates and are expressed as mg Gallic acid Equivalent per g Extract (mgGAE/g). 

### 2.3. Antioxidant Activity

The DPPH (2,2-diphenyl-1-picrylhydrazyl) assay was used to determine the antioxidant activity of different bark extracts. The diluted concentration of 1 mg/mL (solid content) in a volume of 30 µL was filled in a test tube and mixed with 3 mL of the DPPH solution (6 × 10^−5^ M in methanol). The mixture was incubated for 15 min protected from light. Thereafter, the absorbance was measured with a spectrophotometer (Shimadzu UV mini 1240 UV/VIS) at a wavelength of 515 nm. Antioxidant activity was calculated by following formula (as % inhibition).

Inhibition = ((A0 − A15)/A0) × 100, whereby A0 is the blank value and A15 is the measured absorbance at the wavelength 515 nm. The results were calculated from triplicates and are expressed as % inhibition of DPPH at a fixed antioxidant concentration for all the samples.

### 2.4. HPLC Analysis

High-Pressure Liquid Chromatography (HPLC) was performed on an LC-20AT system from Shimadzu (Nakagy-Ku, Kyōto, Japan) equipped with an SPD-M20A photodiode array detector, a CTO 10 AS VP column oven, a Shim-pack C18 column (5 µm, 250 × 4.6 mm) and RID 20 A. The eluent solvents were 0.01% trifluoroacetic acid in water (eluent A) and methanol (eluent B). The mobile phase was an isocratic flow of 80:20 (A:B) at a flow rate of 1 mL/min. In addition, the detector temperature was at 40 °C and the sample injection volume was 20 µL. The flowing rate was 1 mL/min and the detection wavelength was 280 nm. Prior to injection, samples were dissolved in 20% MeOH. As reference substances, DHBA (dihydroxybenzoic acid), HBA (hydroxybenzoic acid), SA (syringic acid), CA (coumaric acid), FA (ferulic acid), C (catechin), EA (ellagic acid), GA (gallic acid) V (vanillin) and VA (vanillin acid) were applied.

### 2.5. Microorganisms Tested

To test the potential antimicrobial activities of the bark compounds, 4 bacterial strains were investigated. All strains were purchased from the American Type Culture Collection (ATCC^®^; Dartford, England) and included *Cutibacterium acnes* (ATCC^®^11827, Gram-positive bacterium, anaerobic), *Staphylococcus epidermidis* (ATCC^®^12228, Gram-positive bacterium, aerobic), MRSA-*Staphylococcus aureus* subsp. *aureus Rosenbach* (ATCC^®^43300, Gram-positive bacterium, aerobic) and *Escherichia coli* (ATCC^®^8739, Gram-negative bacterium, aerobic).

### 2.6. Inoculum Preparation

Bacterial inoculum was prepared from overnight cultures of *E. coli*, MRSA, and *S. epidermidis*. Bacteria were grown on Trypticase Soy Agar (TSA) at 35 °C for 18–20 h. *C. acnes* was cultured at 35 °C for 72 h on Trypticase Soy Agar supplemented with sheep blood (5%) (TSS) (Becton Dickinson, Heidelberg, Germany) at anaerobic conditions (BD GasPak EZ Container System, Becton Dickinson, Sparks, USA). For the Agar Diffusion test, a Mc Farland standard of 0.5 (+/−0.02) according to ~1.5 × 10^8^ CFU of *E. coli* was prepared in sterile, endotoxin-free NaCl 0.9% (Fresenius Kabi, Bad Homburg, Deutschland) for all strains except for the slow-growing anaerobe *C. acnes*, where a McF of 3.0, as also described by Schuster et al. (2020), was used. For the broth microdilution assay, a McF of 0.5 for all test organisms was prepared.

### 2.7. Agar Diffusion Assay

For the initial assessment of antimicrobial activity of certain bark extracts, agar diffusion assays were performed. Blank discs (Oxoid, Basingstoke, UK) were incubated with 100 µL of 10 mg/mL bark extracts overnight. The prepared inocula of different bacterial strains were evenly distributed with sterile cotton swabs on Mueller Hinton E agar (MHE) or for *C. acnes* on Mueller Hinton 2 agar, supplemented with 5% sheep blood (MHF) (Biomerieux, Marcy-l’Étoile, France). Discs containing bark extracts were placed on the inoculated agar plates with sterile tweezers. As positive controls, bacteria-specific antibiotic discs (Oxoid, Basingstoke, UK) were used for aerob bacteria, and Etest^®^ (Biomerieux, Marcy-l’Étoile, France) was used for *C. acnes*. The agar plates were incubated at 35 °C for 20 h, *C. acnes* under anaerobic conditions (BD GasPak EZ Container System, Becton Dickinson, Sparks, NJ, USA) with extended incubation of 24–28 h until visible growth could clearly be observed. Diameters of inhibition zones were measured with a ruler and pictures were taken.

### 2.8. Resazurin-Based Broth Microdilution Assay

For the broth microdilution assay, the prepared inoculum with a McF of 0.5 for all tested strains in NaCl was further diluted in Mueller Hinton Broth (MHB) (Becton Dickinson, Le Pont de Claix, France) or for *P. acnes* in Thioglycolate Broth (Merck KGaA, Darmstadt, Germany) to final bacterial concentrations of ~1 × 10^5^, ~10,000 and ~1000 CFU/mL. A volume of 100 µL of each concentration was seeded in triplicates in 96-well plates (CytoOne^®^, Starlab, Germany) for each bark extract concentration used. Bark extracts were brought to room temperature and added immediately after seeding at 16.67% (*v*/*v*). *E. coli* and MRSA were treated with concentrations of 200 µg/mL, 400 µg/mL and 600 µg/mL extracts. *S. epidermidis* was treated with 100 µg/mL, 150 µg/mL and 300 µg/mL extract concentration. *C. acnes* was treated with concentrations of 25 µg/mL, 50 µg/mL and 100 µg/mL. Aerobic growing bacteria were incubated overnight at 35 °C. *C. acnes* was cultured for 48 h at 35 °C anaerob by using BD GasPak EZ Container System. To quantify the number of metabolically active bacteria, resazurin assay was used. Sterile filtered resazurin sodium salt (Alfa Aesar, Thermo Fisher, Germany) was diluted in PBS w/o Ca and Mg (pH 7.4) to a final concentration of 0.2 mg/mL. After incubation of the microdilution plates, 9.09% (*v*/*v*) of the dye was added to each well and incubated for 45–60 min at 35 °C under aerobic conditions for all strains. Fluorescence signal of enzymatically converted resorufin was detected with Tecan Infinite 200 (560 Ex/610 Em).

### 2.9. Preparation of Heat-Killed C. acnes

Cell lines were stimulated with heat-inactivated *C. acnes* to initiate an inflammatory response. *C. acnes* was chosen since this anaerobic bacterium normally occupies the hair follicles and sebaceous glands and is involved in a number of dermatological conditions. The bacterial suspension was harvested as described in inoculum preparation and centrifuged 800× *g* for 20 min. The pellets were diluted in sterile PBS to a concentration of 1 × 10^10^ CFU/mL and incubated at 80 °C 300 rpm for 30 min. The suspension was stored at 4 °C. To confirm heat inactivation, the bacterial suspension was plated on a TSS plate and incubated for 72 h under anaerobic conditions.

### 2.10. Cell Culture

HaCaT and THP-1 cell lines were purchased from Cell Lines Service in Germany. As recommended by the vendor, HaCaT keratinocytes were grown in DMEM high glucose (4.5 g/L) with stable L-glutamine (2 mM), supplemented with 10% FBS and 1% P/S (Penicillin G Sodium 107 Units/l Streptomycin Sulfate 10,000 mg/L). This medium is referred to as a growth medium. THP-1 monocytes were grown in RPMI 1640 supplemented with 10% FBS, 1% L-glutamine 200 mM and 1% P/S. Both cell lines were maintained at 37 °C in a humid atmosphere with 5% CO_2_. Untreated control samples (UT) contained sterile H_2_O instead of bark extracts.

### 2.11. Cell Viability Assay

To determine the effects of the bark extracts on cell viability, the resazurin sodium salt-based assay was used. Cells were plated in 96-well microtiter plates (CytoOne^®^, Starlab, Germany) at a density of 5 × 10^4^ cells/mL for THP-1 and 2 × 10^5^ cells/mL for HaCaT cells. Both cell lines were incubated overnight at 37 °C in 5% CO_2_. Thereafter, 20 µL of the different bark extracts were applied to the cells to final concentrations of 25, 50, 100, 500 and 1000 µg/mL. Cells were incubated with bark extracts at 37 °C in 5% CO_2_ for 24 h. The cell viability assay was performed by adding 10% (*v*/*v*) resazurin sodium salt at a concentration of 0.2 mg/mL. After 3 h of further incubation, the fluorescence signal of enzymatically converted resorufin was detected with Tecan Infinite 200 (560 Ex/610 Em).

### 2.12. ELISA

For determination of cytokine production of cells treated with heat-inactivated *C. acnes* and various concentrations of bark extracts ELISA analysis was performed. Cells were cultured and treated as previously described for the Cell Viability Assay with resazurin sodium salt. After incubation overnight for 24 h, 96-well plates were centrifuged at 500× *g* for 10 min. Supernatant was immediately used for IL-8 (Immunotools, Friesoythe, Germany) and IL-1beta (Invitrogen, Thermo Fisher Scientific, Bender MedSystems GmbH, Vienna, Austria) ELISA analysis. Both test systems were performed as recommended by the manufacturer’s protocol.

### 2.13. Scratch Assay

The wound-healing assay was performed using 2 well silicone inserts with a 500 µm cell-free gap (Ibidi, Gräfelfing, Germany). HaCaT cells were seeded in 12-well plates at a density of 9 × 10^5^ cells/mL in DMEM growth medium containing 10% FBS in inserts. According to manufacturer’s protocol, 70 µL was applied in every well. To reach confluency, cells were incubated overnight at 37 °C in 5% CO_2_. Thereafter, inserts were removed with sterile tweezers. First, 990 µL DMEM growth medium was applied to the cells, then 10 µL bark extracts diluted in H_2_O was added to final concentrations of 25, 50 and 100 µg/mL. Cells were imaged immediately, and after 24 h and 48 h. Gaps were analyzed using TScratch.

### 2.14. Statistical Analysis

For statistical analysis two-way ANOVA using Dunnett’s multiple comparisons test (for analysis of scratch assay) and Tukey’s multiple comparison test (for analysis of the inhibition zones, viability assay of bacteria and cell lines and ELISA) were performed. Statistics were performed using GraphPad Prism 8.3.0. 

## 3. Results

### 3.1. Extract Characterization

#### 3.1.1. Total Phenolic Content and Antioxidant Activity

Total phenolic content and antioxidant activity of birch, beech and larch bark were analyzed. In addition, crude extracts were compared against sterile filtered extracts to assess the impact of centrifugation and filtration of used extracts. The results of the sterile filtered extracts are depicted in Table 1.

Generally, the crude extracts have a 4.1% higher total phenolic content and a 0.9% lower antioxidant activity than sterile filtered extracts. The marginal difference is supposed to have no influence on the anti-oxidative activity. Nevertheless, the different species have different phenolic content and anti-oxidative potential. 

#### 3.1.2. High-Pressure Liquid Chromatography (HPLC) Analysis

**Figure 1:**
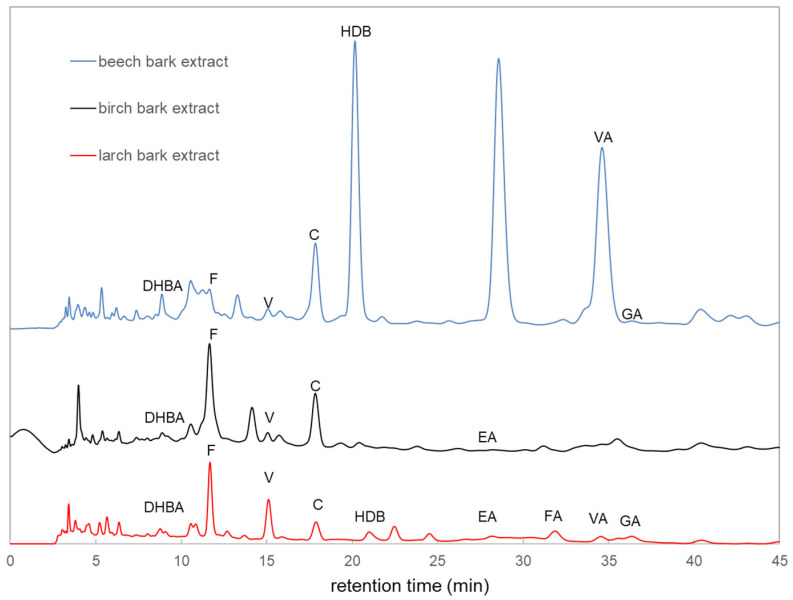
Chromatograms of bark extracts from larch, birch and beech trees. Different substances of DHBA (dihydroxybenzoic acid), HBA (hydroxybenzoic acid), F (unknown flavonoids), V (Vanillin), C (catechin), EA (ellagic acid), FA (ferulic acid), VA (vanillin acid) and GA (gallic acid) were determined.

To gain more insight into the contents of bark extracts, HPLC analysis was performed. Chromatograms taken at 280 nm using a diode array UV/vis detector are presented in Figure 1. The detected components in the different bark extracts are indicated in Figure 1. The HPLC analyses were performed by using various reference substances. The peaks were identified by matching retention time and/or UV spectrum of the labeled corresponding compound, family or molecules, or isomer.

### 3.2. Antimicrobial Activity of Birch, Beech and Larch Extracts

#### 3.2.1. Agar Diffusion Test

Initially, the antimicrobial activity of bark extracts was tested via the agar diffusion method and the inhibition zones of agar plates were measured. All tested Gram-positive strains, including MRSA, *C. acnes* and *S. epidermidis* were susceptible to birch and beech extracts (*p* < 0.0001) compared to negative control with a comparable inhibition zone between 7.5 and 10.5 mm, whereas Gram-negative *E. coli* was resistant to all tested extracts. *C. acnes* was the only tested bacterium with inhibition zones around larch extract discs (7.75 mm, *p* < 0.0001). 

#### 3.2.2. Broth Microdilution Assay

**Figure 2:**
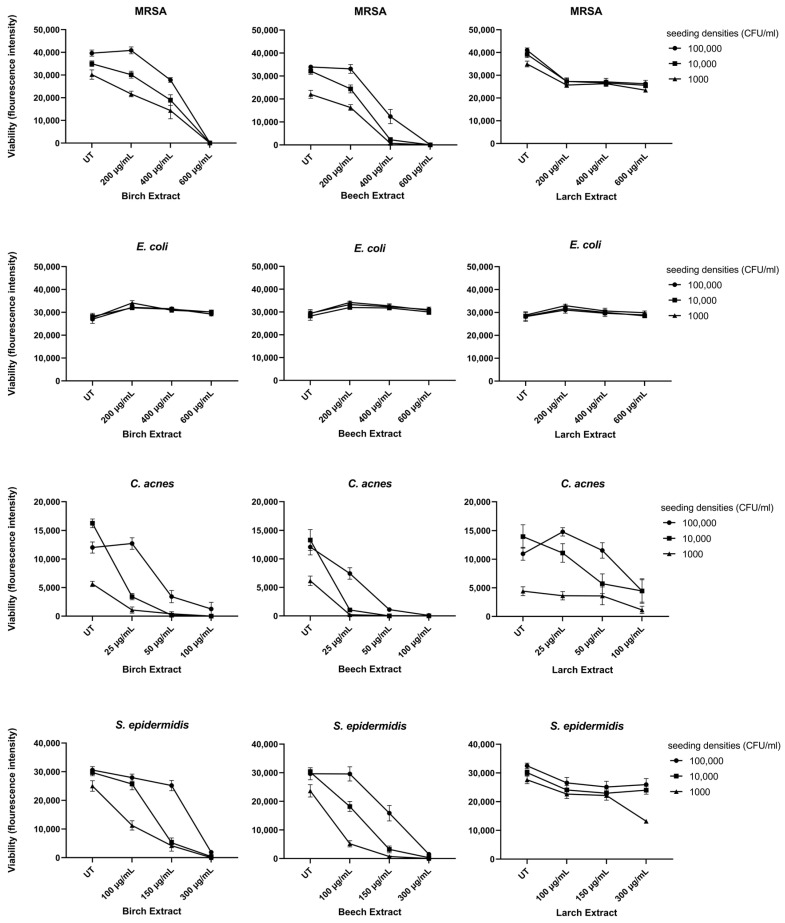
Resazurin viability assay results of the 4 bacterial strains; results are displayed as fluorescence intensity (560 Ex/610 Em).

To further investigate the antimicrobial activity of the bark extracts, a resazurin-based broth microdilution assay was performed. The concentration-dependent reduction in bacterial growth at three seeding densities (100,000, 10,000 and 1000 CFU/mL) upon variable bark extract treatments is shown in Figure 2. The concentrations of bark extracts used in this assay differed depending on the sensitivity of each bacterial strain ranging from 25 to 100 µg/mL for *C. acnes,* 150 to 300 µg/mL for *S. epidermidis* and 200 to 600 µg/mL for MRSA. 

The birch and beech extracts led to a significant reduction in the viability of MRSA, *C. acnes* and *S. epidermidis* compared to the untreated control (UT). Larch extract led to a slight, but significant, reduction in the viability of MRSA, *C. acnes* and *S. epidermidis* compared to the UT; although, no dose-dependent reduction could be observed except for *C. acnes*. In line with the results from the agar diffusion test, Gram-negative *E. coli* showed no susceptibility to any of the extracts tested (Figure 2 and Table 2).

In the broth microdilution assay, resazurin was reduced to resorufin by viable bacteria. High seeding densities of *C. acnes* led to a further reduction of the dye to non-fluorescent dihydroresorufin. Therefore, the UT sample showed less fluorescence intensity at the highest seeding densities. 

### 3.3. Cellular Assays

We further investigated the capacity of the most promising bark extracts, birch and beech, to accelerate wound-healing and regulate the inflammatory response. In order to study these two processes, the HaCaT and THP-1 human cell lines were used. Cell lines were stimulated with heat-inactivated *C. acnes* to initiate an inflammatory response.

#### 3.3.1. Viability Assay

**Figure 3:**
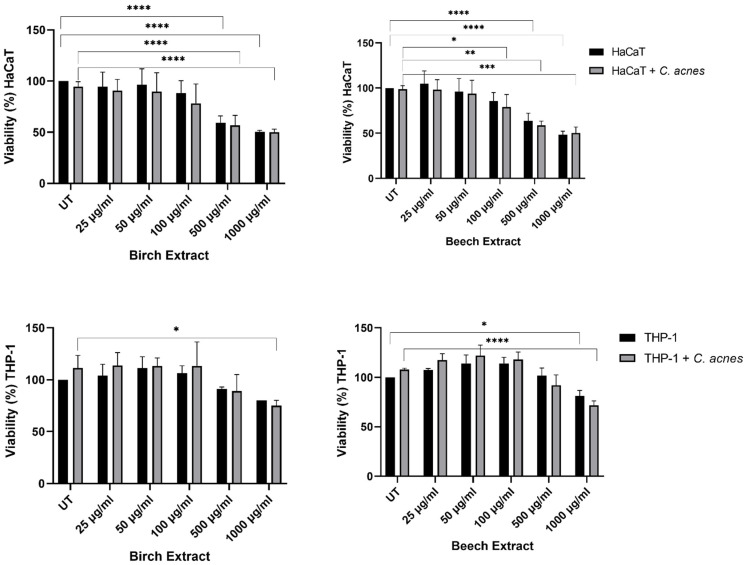
Viability test of HaCaT and THP-1 cell lines upon birch and beech extract treatment shown as percentage compared to the untreated control (UT). Experiments were performed in triplicates, *n* = 3, error bars on column depict SD. HaCaT unstimulated birch and beech: UT vs. 500 µg/mL *p* **** and 1000 µg/mL *p* ****; HaCaT stimulated birch: UT vs. 500 µg/mL *p* *** and 1000 µg/mL *p* ****; HaCaT stimulated beech: UT vs. 100 µg/mL *p* *, 500 µg/mL *p* ** and 1000 µg/mL *p* ***; THP-1 unstimulated beech: UT vs. 1000 µg/mL *p* *; THP-1 stimulated beech: UT vs. 1000 µg/mL *p* ****. Statistical significance: * *p* < 0.05, ** *p* < 0.01, **** *p* < 0.0001.

To ensure a non-toxic dose of bark extracts for all cell-based assays, we investigated the toxic potential of the bark extracts using resazurin viability assays in the HaCaT and THP-1 cell lines unstimulated and stimulated with *C. acnes*. Concentrations up to 100 µg/mL did not significantly reduce the viability in HaCat cells, except for the beech extract at 100 µg/mL in stimulated HaCaT cells (Figure 3) compared to UT. The highest concentrations (500 and 100 µg/mL) of beech and birch extracts led to a significant decrease in the viability of the HaCaT cells (Figure 3) compared to UT.

THP-1 cells showed a non-significant increase in viability upon bark extract treatment up to 500 µg/mL. Higher concentrations resulted in a significant decrease (Figure 3) compared to UT. The heat-inactivated *C. acnes*-stimulated samples did not differ significantly from native samples in both cell lines.

#### 3.3.2. Pro-Inflammatory Cytokine Measure

To gain more insight into proinflammatory signaling in response to birch and beech extract treatment, ELISA analysis was performed from cell culture supernatants, either native or stimulated with heat-inactivated *C. acnes.*

**Figure 4:**
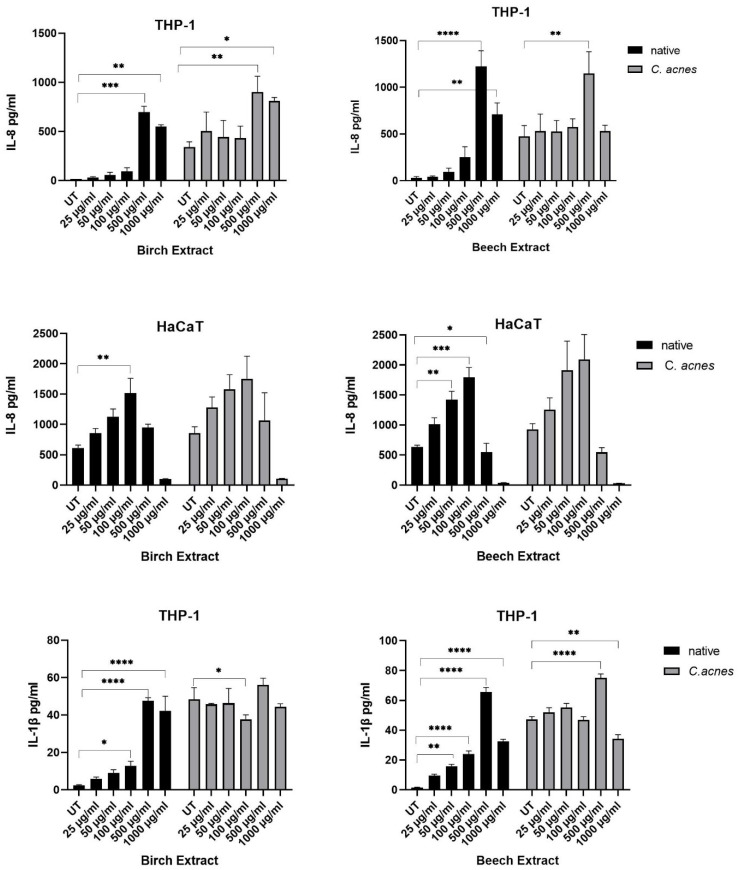
Pro-inflammatory cytokine expression (IL-8 and IL1beta) in THP-1 and HaCaT cell supernatants after 24 h incubation with extracts. Columns present means of triplicates (*n* = 3); the error bars show SD. * *p* < 0.05, ** *p* < 0.01, *** *p* < 0.001, **** *p* < 0.0001.

In THP-1 cells, a dose-dependent increase in IL-8 expression was seen up to 500 ug/mL of birch and beech extract in native and stimulated cells compared to UT (Figure 4). The decrease in IL-8 expression at 1000 µg/mL of birch and beech extract correlates with a significant decrease in viability as seen in Figure 3. The baseline production of IL-8 was generally higher in stimulated samples compared to native samples.

The results from HaCaT supernatants showed a basal production of IL-8 of around 600 pg/mL in unstimulated, untreated samples and around 900 pg/mL in *C. acnes*-stimulated samples. The IL-8 production increased with bark extract concentrations up to 100 µg/mL, concentrations above this showed a significant decrease in IL-8 release in line with the cell viability results. The release of IL-1β in response to bark extract treatment was confirmed for THP-1 but not for HaCaT cells (data not shown). Concentrations between 25 and 500 µg/mL of birch and beech extracts led to a dose-dependent increase in IL-1β in native THP-1 monocytes (Figure 4). Baseline concentrations were higher in stimulated samples, again the highest level of IL-1β was measured at 500 µg/mL of birch and beech extracts. Concentrations of 1000 µg/mL in both extracts showed a decreased IL-1β release correlating to the viability results.

#### 3.3.3. Scratch Assay

**Figure 5:**
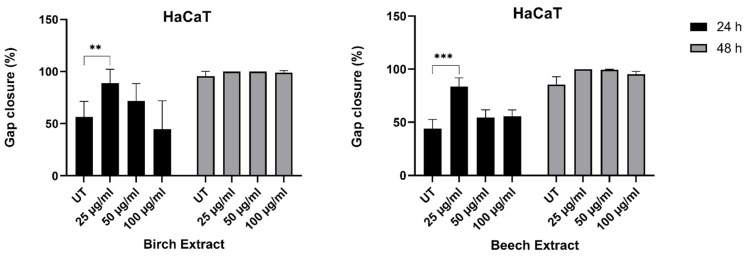
Wound-healing capacity of birch and beech extract reported as percentage gap closure at 24 and 48 h. The gap was defined as 0% closed at the beginning of the experiment. A fully closed gap resulted in 100% closure. N = 4, error bars present SD. Statistical significance: ** *p* < 0.01, *** *p* < 0.001.

For testing the wound-healing capacity of birch and beech extracts, Scratch Assays were performed and closure of the gap at 24 and 48 h was reported. Birch and beech extracts at a concentration of 25 µg/mL significantly accelerated the wound closure measured after 24 h, with 88.9% and 83.7% wound closure in the birch and beech extracts compared to 56.3% and 43.8% in the respective untreated control sample (Figure 5).

Higher doses of 50 µg/mL and 100 µg/mL showed a minor, non-significant effect in accelerating wound closure. After 48 h the gaps were closed in all samples.

## 4. Discussion

In this study, we focused on the use of larch, birch and beech bark extracts from by-products of the wood industry to investigate antimicrobial properties, wound-healing capacities and the inflammatory mode-of-action to identify the potential of these extracts for dermatological applications. Antibacterial properties of various wood and bark extracts have already been described in a number of studies [6,7,8,9,21,22]. Nevertheless, information regarding the use of tree extracts in biocompatible solvents for biological applications, especially for beech bark, is limited.

To assess the antimicrobial activity, common skin-related bacteria that can cause dermatological disorders were chosen. All tested Gram-positive strains, including MRSA, *C. acnes* and *S. epidermidis,* were susceptible to birch and beech extracts, whereas Gram-negative *E. coli* was resistant to all tested extracts. Preliminary experiments in our lab with multiple Gram-negative bacteria including *P. aeruginosa, S. typhi and K. pneumoniae* (data not shown) showed no or low response to bark extract treatment compared to Gram-positive species. Recent studies, focusing on the antimicrobial activity of wood and bark extracts, observed that Gram-positive bacteria are more sensitive to wood and bark extracts than Gram-negative bacteria and can be inhibited by a wider range of wood and bark extracts. [9,22] The difference between the Gram-negative vs. Gram-positive bacteria can be explained by the difference in their outer structure, since Gram-negative bacteria harbor an additional outer membrane compared to a thick peptidoglycan layer in Gram-positive bacteria. Interestingly, larch bark extracts only inhibited the growth of *C. acnes,* which suggests a different mode of action compared to birch and beech extracts. The antibacterial effect of larch bark has been linked to the content of procyanidins, which significantly alters cell wall integrity and membranes as well as protein synthesis and can form complexes with DNA in *S. aureus* [23]. Further studies are required to assess if procyanidins are involved in the growth inhibition of *C. acnes* and how this mechanism differs compared to other bacterial strains.

The phenolic compounds, present in wood and bark extracts are known to have variable antimicrobial activities [7,24]. Phenolic compounds disrupt cell membranes, and are able to inhibit DNA gyrase or protein kinases. The antibacterial effect can strongly be enhanced by synergistic action, which has been observed for many phenolic compounds. [25] Hydroxybenzoic acids, present in all tested bark extracts, are known for their activity against bacteria including *E. coli* and *S. aureus* [26]. Interestingly, in our study, no inhibition of *E. coli* was observed even though hydroxybenzoic acid was present. This difference could be explained by the actual amount of hydroxybenzoic acid in an extract in comparison to chemically synthesized parabens as a sole inhibitory agent. Furthermore, phenolic acids, including hydrobenzoic acids, show antioxidant capacities [27,28]. Our findings confirm that extracts with the highest phenolic content showed the highest antioxidant activity. Based on different literature results from the HPLC analyses, the chemical composition of the bark extracts is quite complex [8,29]. However, the antioxidant efficiency of beech bark phenols is dependent on only a few substances [29]. In this current study, additional substances could be detected using the hot water extraction method compared to the results of beech bark from Tanase et al. [30]. Tanase et al., used a microwave extraction method and found the same substances, such as vanillin acid, gallic acid, catechin and other flavonoids. However, we found also different isomers of DHBA (dihydroxybenzoic acid) and HBA (4-Hydroxybenzoic acid). These substances were detected in samples of wood condensates from industry [31]. By using hot water extraction methods, the change of bark components cannot be excluded through different reaction processes due to the effect of high temperature and longer procedure time compared to other methods. Frequently, conversion processes occur here in the carbohydrates (polyoses) to hydroxymethylfurfural or other chemical modifications.

In addition to the mode-of-action of phenolic compounds, the rate of bacterial growth influences the extent of inhibition. MRSA is a rapidly growing bacteria with a doubling time of 20 min under optimal conditions [32]. The concentration for full growth inhibition was 600 µg/mL in birch and beech extracts. It has been observed that slower-growing bacteria could be inhibited with much lower concentrations. *S. epidermidis* was fully inhibited with half of the concentration and is known to have a doubling time of 55 min [33]. For *C. acnes,* this dose was 100 µg/mL. The slow-growing anaerobia have a 3.5 h doubling time under optimal laboratory conditions. *C. acnes* is a fastidious bacterium and generation time is strongly diminished under suboptimal conditions including changes in pH, oxygen tension and carbon sources. The presence of 20% oxygen causes a doubling time of 17.25 h. [34] Therefore it is supposed that much lower concentrations of bark extracts could suppress growth in the natural habitat of follicles on the human skin.

To further investigate the potential of bark extracts for dermatological applications, a wound-healing assay was performed using the human cell line HaCaT keratinocytes. In addition to the HaCaT cell line, a monocytic THP-1 cell line was used to study the inflammatory response after exposure to bark extracts. Larch extract showed minor effects in antimicrobial activity and was therefore excluded in the cell assays. Initially, the viability of both cell lines was measured after bark extract exposure to define a non-toxic extract concentration. HaCaT keratinocytes were more sensitive to high concentrations of bark extracts than the monocytic THP-1 cell line. In the wound healing experiment, 25 µg/mL of bark extracts significantly accelerated the wound closure and increasing levels of bark extracts showed enhanced secretion of the proinflammatory cytokines IL-8 and IL-1β after 24 h incubation. Even though activation of the immune response is part of a wound healing process, excessive inflammation counteracts the wound-healing mechanism [35]. The lack of accelerated wound closure at higher concentrations could be explained by the extent of inflammatory cytokine secretion, which occurred only at higher concentrations.

Recent studies showed that triterpene extracts and betulin from birch significantly accelerate wound closure [16], which can explain the results observed in our study even though the amount of triterpene was not quantified. Regarding the positive effect of beech bark extracts on cell migration, very little is known about the chemical compounds responsible for the accelerated closure and further studies are needed to elucidate this effect.

The combined properties of antimicrobial activity and wound healing capacities make bark extracts extremely attractive for various applications on the human skin [36]. There is great potential for numerous treatments in dermatology, including *acne vulgaris*. It can be used as additives in soaps, shampoos or creams for increasing shelf life on the one hand and protecting the skin on the other. Additionally, these products may prevent small superficial skin injuries from infections and help them heal faster [37].

In terms of the sustainable use of by-products from the wood industry, the use of extracts from the bark of local trees poses a promising way to maximize the utility of these currently underutilized or discarded products [38]. Consumers are increasingly turning towards the use of natural alternatives in cosmetics, cosmeceuticals and medicine, which is in line with the increasing awareness of the sustainable management of natural resources. Although the use of phytochemicals in various dermatological disorders is promising, there are still limited numbers of clinical trials in humans [39]. Thus, research in the area of phytochemicals for medical use is of utmost importance to advance the number of phytochemical-based drugs.

## 5. Conclusions

Our findings show a successful growth inhibition with birch and beech bark extracts of Gram-positive bacteria, involved in skin diseases, such as acne. Wound closing using an in vitro scratch assay was shown to be faster with low concentrations of birch and beech extract treatments compared to controls. Whereas beneficial properties of birch components are already described in the literature, the very similar effects of beech contents are new. Their combined positive effect on wound-healing and antimicrobial activity has great potential for the treatment of various skin diseases. In addition, the use of biomolecules in the form of an extract from bark, which is often considered as waste or a by-product from a variety of industrial processes, enables the sustainable use of natural and renewable products.

## Figures and Tables

**Table 1:** Total phenolic content in GAE/g and antioxidant activity of sterile-filtered bark extracts.

	Total Phenolic Content (mg GAE/g)	Antioxidant Activity (%)
**Birch**	440.74	81.94
**Beech**	297.86	50.68
**Larch**	277.62	48.35

**Table 2:** Statistical analysis of resazurin-based broth microdilution assay for each individual seeding density with different extracts compared to UT.

		**Birch µg/mL**			**Beech µg/mL**			**Larch µg/mL**		
	CFU/mL	**200**	**400**	**600**	**200**	**400**	**600**	**200**	**400**	**600**
**MRSA**	100,000	ns	****	****	ns	****	****	****	****	****
	10,000	ns	****	****	**	****	****	****	****	****
	1000	**	****	****	*	****	****	****	****	****
***E. coli***	100,000	***	**	ns	ns	ns	ns	ns	ns	ns
	10,000	*	ns	ns	ns	ns	ns	ns	ns	ns
	1000	****	ns	ns	*	ns	ns	ns	ns	ns
	CFU/mL	**25**	**50**	**100**	**25**	**50**	**100**	**25**	**50**	**100**
***C. acnes***	100,000	ns	****	****	***	****	****	ns	*	*
	10,000	****	****	****	****	****	****	ns	***	****
	1000	****	****	****	****	****	****	ns	ns	ns
	CFU/mL	**100**	**150**	**300**	**100**	**150**	**300**	**100**	**150**	**300**
***S. epi* ^1^**	100,000	ns	*	****	ns	****	****	*	**	*
	10,000	ns	****	****	****	****	****	*	**	*
	1000	****	****	****	****	****	****	ns	ns	****

^1^*S. epidermidis*; Statistical significance: * *p* < 0.05, ** *p* < 0.01, *** *p* < 0.001, **** *p* < 0.0001.

## Data Availability

Data are available on request from the corresponding author.

## Sample Availability

Samples of the compounds are not available from the authors.
